# Advanced Energy Storage Devices: Basic Principles, Analytical Methods, and Rational Materials Design

**DOI:** 10.1002/advs.201700322

**Published:** 2017-11-15

**Authors:** Jilei Liu, Jin Wang, Chaohe Xu, Hao Jiang, Chunzhong Li, Lili Zhang, Jianyi Lin, Ze Xiang Shen

**Affiliations:** ^1^ Division of Physics and Applied Physics School of Physical and Mathematical Sciences Nanyang Technological University Singapore 637371 Singapore; ^2^ College of Aerospace Engineering Chongqing University Chongqing 400044 P. R. China; ^3^ Key Laboratory for Ultrafine Materials of Ministry of Education School of Materials Sciences and Engineering East China University of Science and Technology 130 Meilong Road Shanghai 200237 P. R. China; ^4^ Heterogeneous Catalysis Institute of Chemical Engineering and Sciences A*star, 1 Pesek Road Jurong Island 627833 Singapore; ^5^ Energy Research Institute @NTU (ERI@N) Nanyang Technological University Singapore 639798 Singapore

**Keywords:** advanced energy storage devices, analytical methods, pseudocapacitance, rational materials design

## Abstract

Tremendous efforts have been dedicated into the development of high‐performance energy storage devices with nanoscale design and hybrid approaches. The boundary between the electrochemical capacitors and batteries becomes less distinctive. The same material may display capacitive or battery‐like behavior depending on the electrode design and the charge storage guest ions. Therefore, the underlying mechanisms and the electrochemical processes occurring upon charge storage may be confusing for researchers who are new to the field as well as some of the chemists and material scientists already in the field. This review provides fundamentals of the similarities and differences between electrochemical capacitors and batteries from kinetic and material point of view. Basic techniques and analysis methods to distinguish the capacitive and battery‐like behavior are discussed. Furthermore, guidelines for material selection, the state‐of‐the‐art materials, and the electrode design rules to advanced electrode are proposed.

## 1 Introduction

**Figure 1:**
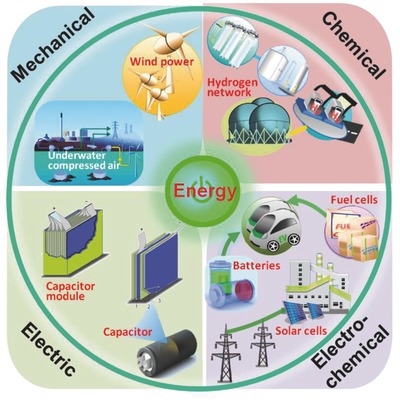
Classification of different types of energy storage technologies for stationary applications.

**Figure 2:**
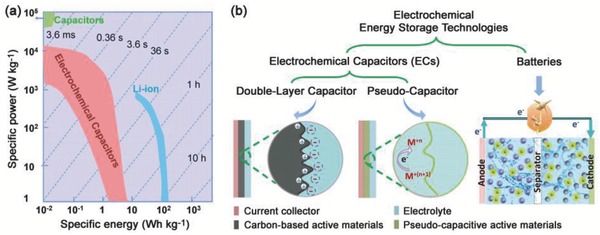
a) Ragone plot comparing the power‐energy characteristics and charge/discharge times of different energy storage devices. b) Schematic diagram comparing the fundamental mechanisms of electrochemical energy storage in double‐layer capacitors, pseudocapacitors, and batteries. Reproduced with permission.^22^ Copyright 2016, The Springer Nature.

Urgent exploitation of renewable and sustainable energy sources, such as wind and solar energy, has been prompted by environmental concerns related to the continuous consumption of nonrenewable resources and the increasing complexity of power distribution systems. Efficient usage of these new energy sources is crucial concerning their nonconstant power generation. Hence, a popular strategy is to develop advanced energy storage devices for delivering energy on demand.^1^, ^2^, ^3^, ^4^, ^5^ Currently, energy storage systems are available for various large‐scale applications and are classified into four types: mechanical, chemical, electrical, and electrochemical,^1^, ^2^, ^6^, ^7^, ^8^ as shown in **Figure** **1**. Mechanical energy storage via pumped hydroelectricity is currently the dominant energy storage method. However, electrochemical energy storage (EES) systems in terms of electrochemical capacitors (ECs) and batteries have demonstrated great potential in powering portable electronics and the electrification of the transportation sector due to the advantageous features of high round‐trip efficiency, long cycle life, and potential to be implemented with various chemistries based on cheap, sustainable and recyclable materials, and low maintenance cost.^1^, ^2^, ^6^ Generally, electric energy is stored in EES in two ways: directly via a non‐faradaic process or indirectly via a faradaic process.^9^ The non‐faradaic technologies store electricity directly in an electrostatic way. Typically, electric double‐layer capacitors (EDLCs) are efficient (≈100%) and suitable for power management (e.g., frequency regulation), but deliver a low energy density with limited discharge time.^10^ Alternatively, electrical energy can be stored by converting it to available chemical energy, requiring faradaic oxidization and reduction of the electrochemically active reagents, and reversibly release the energy on demand. Typical examples of faradaic systems include pseudocapacitors and various batteries. Ragone plot in **Figure** **2**a compares the power and energy relationship of various EES systems. Pike Research forecasted that the grid‐scale stationary EES system revenues will grow from $1.5 billion in 2010 to $25.3 billion over the following ten years, with the most significant growth in EES technologies.^6^, ^11^

While there is significant potential for the application of EES technologies, their operating performance is still lagging behind the increasingly harsh requirements of industry. One key challenge is the identification of ideal electrode materials that satisfy the requirements of high energy/power densities and long cycle.^12^ Strategies such as decreasing the particle size,^13^, ^14^ controlling the morphology,^15^ and producing hybrid materials^16^ have been proposed. Such novel electrode materials reduce the gap in electrochemical behavior between ECs and batteries, mainly because of the popular trend toward increasing the mutual penetration of nanostructured materials (combining the high energy density of batteries with the high power density of pseudocapacitors). For example, the same electrode material may display pseudocapacitive or battery‐like behavior depending on its structure, morphology, particle size, and intercalation ion (i.e., Li^+^ or Na^+^). In addition, the fabrication of hybrid materials that combine two or more electroactive materials in a single‐electrode design increases the complexity of the electrochemical behavior.^17^ Detailed comparisons of EES devices via appropriate measurements and analyses would be helpful to avoid any confusion and inappropriate claims in the field of electrochemical energy storage. Moreover, such an investigation would promote better fundamental understanding and provide basic guidance for material selection and electrode design for high‐performance energy storage devices.

In this review, we first introduce fundamental electrochemistry principles and the basic analysis methods used to identify capacitive features. Based on these general properties we will discuss examples of how pseudocapacitive and battery‐type materials are distinguished and classified. We then introduce the state‐of‐the‐art materials and electrode design strategies used for high‐performance energy storage. Intrinsic pseudocapacitive materials are identified, extrinsic pseudocapacitive materials are discussed, and novel hybrid structures are proposed for high‐performance energy storage devices. This review is expected to contribute to a better fundamental understanding of the electrochemistry and practical analysis methods for characterizing various nanostructured electrode materials for advanced electrochemical energy storage technologies.

## 2 Principle of Energy Storage in ECs

EC devices have attracted considerable interest over recent decades due to their fast charge–discharge rate and long life span.^18^, ^19^ Compared to other energy storage devices, for example, batteries, ECs have higher power densities and can charge and discharge in a few seconds (Figure 2a).^20^ Since General Electric released the first patent related to ECs in 1957,^21^ these devices have been applied in many fields, including power capture and supply, power quality applications, and backup power.^19^

### 2.1 Basics of Double Capacitance and Pseudocapacitance

ECs are classified into two types based on their energy storage mechanisms: EDLCs and pseudocapacitors (Figure 2b).^9^, ^23^, ^24^ In EDLCs, energy is stored via electrostatic accumulation of charges at the electrode–electrolyte interface.^19^ In the case of pseudocapacitors, energy is stored by the electrosorption and/or reversible redox reactions at or near the surface of the electrode material, usually a conducting polymer or transition metal oxide.^18^, ^22^, ^24^, ^25^, ^26^ In general, both these mechanisms exist in a supercapacitor device.

#### 2.1.1 Charge Storage Mechanism in EDLCs

The energy storage of EDLCs is via charge adsorption at the surface of the electrode without any faradaic reactions.^24^, ^27^ During the charge/discharge processes, the arrangement of the charges in the Helmholtz double layer results in a displacement current. Since the materials can respond quickly to the change of potential and the physical reaction in nature, EDLCs can deliver energy quickly, as shown in the Ragone plot in Figure 2a.^23^, ^28^ However, due to the confinement of the electrode surface, the amount of stored energy is limited and much lower than that of pseudocapacitors and batteries. The EDL capacitance is described as follows^9^, ^10^

(1) $$C_{\mathrm{dl}}=\frac{Q}{V}=\frac{\varepsilon_{r}\varepsilon_{o}A}{d}$$

where *C*_dl_ is the EDL capacitance of a single electrode, *Q* is the total charge transferred at potential *V*, ε_r_ is the dielectric constant of the electrolyte, ε_o_ is the dielectric constant of vacuum, *d* is the charge separation distance, and *A* is the electrode surface area.

When *C*_dl_ is constant for EDLCs, the following equation describing the response current *I* can be derived from Equation (1)

(2) $$I=\frac{dQ}{dt}=C_{\mathrm{dl}}\;\frac{dV}{dt}$$

where *t* is the charge time.

If the applied voltage *V* varies with time *t* in a linear way, that is, *V* = *V*_0_ + *vt* (where *V*_0_ is the initial voltage and *v* is the sweep rate (V s^−1^ or mV s^−1^)), the relationship can be described as

(3) $$I=C_{\mathrm{dl}}\;v$$

**Figure 3:**
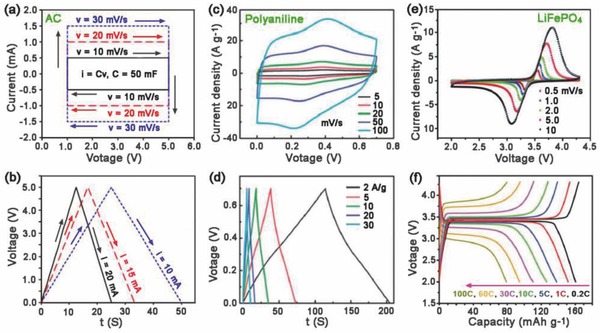
Cyclic voltammograms (top) and galvanostatic charge/discharge curves for different types of electrode materials. a,b) Carbon‐based double‐layer supercapacitor. Reproduced with permission.^49^ Copyright 2013, Chinese Materials Research Society. c,d) Polyaniline pseudocapacitive electrode. Reproduced with permission.^50^ Copyright 2013, The Royal Society of Chemistry, and e,f) LiFePO_4_ battery electrode (vs Li). Reproduced with permission.^51^ Copyright 2015, The Royal Society of Chemistry. These series show a wide range of sweep rates and current densities, highlighting the unique electrochemical features of each material. A transition from (a, b) typical capacitive behavior to (e, f) typical battery behavior has been well illustrated with (c, d) pseudocapacitive behavior as an intermediate case.

The current responds linearly with the sweep rate, as shown in Equation (3). This translates into a well‐defined rectangular current (*I*)–voltage (*V*) plot or cyclic voltammogram for different sweep rates (**Figure** **3**a). Alternatively, if the capacitor is charged or discharged under a constant current, the voltage will increase (charging) or decrease (discharging) with a constant rate, as calculated by Equation (3). Thus, a triangular charge/discharge curve is expected, as shown in Figure 3b.

Over the past decades, significant progresses have been made in fundamental understanding and design of electrode materials for energy storage devices. Carbon‐based materials, such as activated carbons (ACs),^29^, ^30^ carbon nanotubes (CNTs),^31^ and graphene,^32^, ^33^ are regarded as EDLC supercapacitors, where their electrode surface area and surface state,^34^, ^35^, ^36^ pore structure and pore size distribution,^29^, ^37^, ^38^ and number of carbon layers,^39^ are critical parameters. To date, great efforts have been made to improve the energy density of EDLCs, considering matching carbon pore sizes with the electrolyte ion size,^38^ oxygen functionalizing the carbon surface^40^ or tailoring the oxygen content,^41^ modifying carbon with heteroatom (N, S, F, etc.) doping^42^, ^43^, ^44^ or co‐doping,^45^, ^46^ adopting redox active species‐based electrolytes,^47^ and designing ionic liquids with high working voltage and a wide temperature range.^48^ However, EDLC supercapacitors can still not meet the rigid requirement for high‐energy density devices due to intrinsic drawbacks, limiting their large‐scale application.

#### 2.1.2 Transition from Electrophysical Storage to Pseudocapacitive Storage

Pseudocapacitance is a faradaic energy storage based on the fast redox reaction on the surface or near‐surface region of the electrodes, where electrosorption/electrodesorption occurs with charge transfer but without any bulk phase transformation upon charging/discharging (Figure 2b).^26^, ^52^, ^53^ The state of charge (*q*) is a function of the electrode potential with the extent of faradaic charge/discharge (*Q*) passed.^24^ The change in *Q* with respect to the potential gives rise to the derivative, d*Q*/d*V*, which corresponds to the pseudocapacitance (*C*_∅_).^26^ Unlike EDL capacitance, which is associated with potential‐dependent accumulation of electrostatic charge (Figure 3a,b), pseudocapacitance is faradaic in nature (Figure 3c,d).^24^, ^26^, ^54^ In addition, there are differences between pseudocapacitance and the ideal Nernstian process involved in battery‐type materials where faradaic reactions occur at a constant potential (Figure 3e,f).^54^

**Figure 4:**
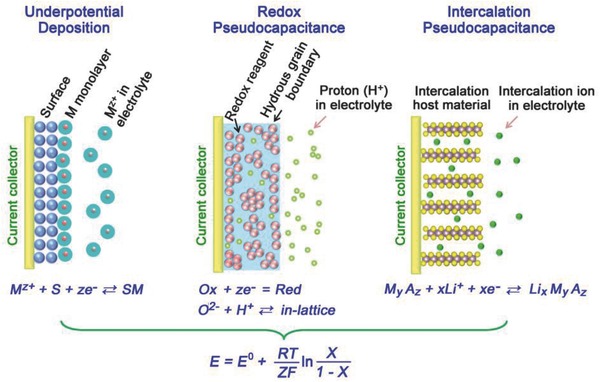
Schematic diagrams of the different faradaic processes that give rise to pseudocapacitance. Here, X is the 2D site occupancy fraction for underpotential deposition, [O_X_]/([O_X_] + [Red]) for redox systems and the occupancy fraction of layer lattice for intercalation systems, respectively. Reproduced with permission.^22^ Copyright 2016, The Springer Nature.

According to Conway et al., pseudocapacitance can be classified into three types: (i) underpotential deposition (UPD) (2D), (ii) surface redox system (2D), and (iii) intercalation system (quasi‐2D), as shown by the schematic diagrams in **Figure** **4**.^24^, ^26^

When a potential is applied to a metal, an adsorbed monolayer will form on the surface due to the reduction of a different metal ion, resulting in a less negative potential than their equilibrium potential; this process is referred to as UPD.^55^, ^56^ The deposition of Pb on Au is a typical example of UPD.^52^, ^57^ As the Pb—Au interaction is stronger than that of Pb—Pb in crystalline Pb metal, Pb can deposit onto Au more easily than onto itself. UPD can be applied both to metal deposition and other adsorbed layers, for example, H from H_3_O^+^ or H_2_O pseudocapacitance.^58^

The redox system is a typical form of pseudocapacitance, where the mechanism involves the adsorption of electroactive ions onto the surface or near‐surface region of electrode materials and faradaic reactions occur with charge transfer. Typical examples include transition metal oxides (e.g., RuO_2_ 59, 60 and MnO_2_ 61, 62) and conducting polymers generated using electrochemical methods (e.g., polyaniline (PANI),^63^, ^64^ polypyrrole,^65^, ^66^ and poly(3,4‐ethylenedioxythiophene)^67^, ^68^). Transition metal oxides exhibit pseudocapacitance on the bias of fast redox reactions caused by the intercalation of protons (H^+^) or alkali metal cations (C^+^ = Na^+^, K^+^, etc.), as described below^69^, ^70^

(4) $${\mathrm{MO}}_{2}+H^{+}+e^{-}↔\mathrm{MOOH}$$

or

(5) $${\mathrm{MO}}_{2}+C^{+}+e^{-}↔\mathrm{MOOC}$$

Upon charging/discharging, no chemical transformation occurs, but a reversible functionalized molecular layer forms on the electrode surface due to faradaic reactions. The potential of the electrode has a linear dependence on the charge and is proportional to the area of the electrode surface covered by electroactive ions. These features are completely different from the redox reactions involved in a battery‐type electrode, as mentioned previously.

ECPs can store and release charges through redox processes involving the π‐conjugated polymer chains during electrochemical doping–undoping, as described in the following reaction^71^, ^72^, ^73^

(6) $$\left[\mathrm{ECP}\right]+nX^{-}↔\;\;\left[{\left(\mathrm{ECP}\right)}^{n+}nX^{-}\right]+ne^{-}$$

During the oxidization (p‐doping) process, the anionic species X^−^ from the electrolyte are inserted into the polymer backbone and are released back into the electrolyte upon reduction. The embedding and stripping of counter ions enable high specific capacitance values, similar to a battery‐type reaction. However, ECPs suffer from volumetric changes during such reactions and poor cycling performance is observed due to poor mechanical properties of these inherently brittle materials.^73^ Hence, many efforts have been made to overcome these drawbacks. The most desirable and programmable way is to combine carbon materials (e.g., CB,^74^ CNTs,^75^ or graphene^76^) with ECPs to improve the mechanical properties.

Intercalation pseudocapacitance is another faradaic process occurring without a crystallographic phase change and arising when intercalation sorption of the quasi‐2D electroactive species take place. It differs from the intercalation process involved in a battery where crystallographic phase transformation occurs during the charge transfer processes. Intercalation systems in pseudocapacitors include the intercalation of Li^+^ ions into hosts such as TiS_2_,^57^, ^77^ MoS_2_,^78^, ^79^ and V_6_O_13_ 80 or the intercalation of H into Pd and Pd‐Ag alloys.^52^, ^57^ Recently, novel 2D materials have been developed. Among them, transition metal carbides (MXenes) with the chemical formula M*_n_*_+1_X*_n_*T*_n_* (where M is a transition metal, X is C and/or N, and T*_n_* denotes surface functionalization), are introduced as unique host materials for intercalation pseudocapacitors.^81^, ^82^ High‐volume pseudocapacitors have been developed through the intercalation of ions such as Li^+^, Na^+^, K^+^, NH_4_^+^, or Al^3+^ into Mxene layers. As shown in Equation (7), Ti_3_C_2_T*_n_*, a typical Mxene material, shows high volumetric capacitance values due to changes in the Ti oxidation state during the intercalation/de‐intercalation processes

(7) $${\mathrm{Ti}}_{3}C_{2}O_{x}{\left(\mathrm{OH}\right)}_{y}F_{2-x-y}+\delta e^{-}+\delta H^{+}↔{\mathrm{Ti}}_{3}C_{2}O_{x-\delta }{\left(\mathrm{OH}\right)}_{y+\delta }F_{2-x-y}$$

These three pseudocapacitance mechanisms are based on different faradaic processes and occur in different types of materials; however, they deliver similar thermodynamic features, that is, a logarithmic relationship between the electrode potential and the extent of charge/discharge, as shown in Figure 4, 9, 18, 24, 53

(8) $$E=E^{0}+\frac{RT}{nF}\ln\frac{X}{1-X}$$

where *E* is the electrode potential (V), *R* is the ideal gas constant (8.314 J mol^−1^ K^−1^), *T* is the temperature (*K*), *n* is the number of electrons, *F* is Faraday's constant (96 485 C mol^−1^), and *X* is the occupancy fraction of the surface or lattice layer.

If the electrochemical sorption of electroactive species follows an electrochemical Langmuir isotherm,^52^, ^53^ as shown in Equation (9), the pseudocapacitance is defined as shown in Equation (10),^24^ where q* is the charge required for completion of monolayer sorption:

(9) $$\frac{X}{1-X}=k\cdot \exp\left(\frac{VF}{RT}\right)$$

(10) $$C_{\emptyset }=q^{*}\cdot \frac{dX}{dV}$$

From Equations (9) and (10), it is identified that the pseudocapacitance *C*_∅_ is not constant and has a maximum value at *X* = 0.5. Pseudocapacitors store energy in a similar way to EDL capacitors (where the stored charge is a continuous function of *E*), while the main difference is that pseudocapacitance involves faradaic charge transform. Specifically, for pseudocapacitance, the electrode potential is associated with the conversion of the electroactive material, such as the degree of utilization of free sites on the surface or within the 2D or quasi‐2D material (Δ*G* = Δ*G* ^θ^ + *RT* In(*X*/(1 − *X*)). In the case of most battery electrodes, a certain electrode potential is determined by the Gibbs free energies of pure, well‐defined 3D phases and usually also the composition and/or concentration of the solution (Δ*G* = − *nFE* ^θ^).^4^, ^9^ In addition, pseudocapacitors always show higher rate capability values than batteries benefiting from the surface/near surface reaction (Figure 2).

### 2.2 Kinetic Electrochemical Features of ECs

The difference in charge storage mechanisms leads to different kinetic behavior upon the application of (i) potentiodynamic sweep and (ii) constant current (galvanostatic charge/discharge curves)^18^, ^24^ as shown in Figure 3. These mechanisms will be discussed in the following sections in more detail.

#### 2.2.1 Potentiodynamic Sweep Cyclic Voltammetry (CV)

**Figure 5:**
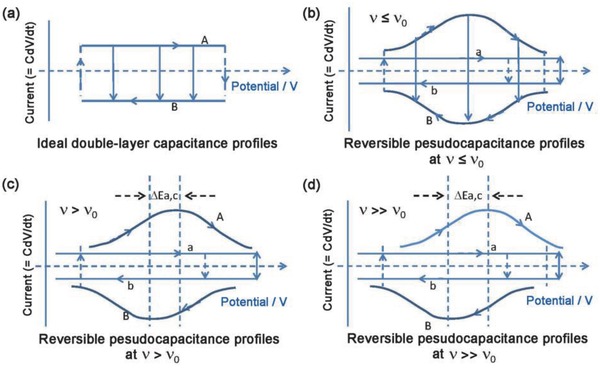
CV profiles of a) ideal double‐layer capacitor and b–d) reversible pseudocapacitors with different sweep rates *v*, where *v*_0_ is the critical sweep rate.^24^, ^83^, ^84^ Reproduced with permission.^24^ Copyright 1991, The Electrochemical Society.

During CV testing, an ideal capacitive system shows symmetric cyclic voltammograms at slow sweep rates, and there is ideally no or only small potential shifts between the anodic and cathode peaks under various sweep rates (**Figure** **5**a,b).^18^, ^24^

With the exception of air and vacuum dielectric capacitors, all ECs have an effective equivalent series resistance (ESR), which results in the polarization effect and deviations from ideal capacitive behavior.^24^ The presence of the charge transfer resistance in pseudocapacitors imposes kinetic limitations. If the surface faradaic processes involved for pseudocapacitors are modulated by an increasing sweep rate (*v*), the kinetic reversibility is gradually lost.^24^ This is manifested by the peak potential, *E*_p_, which remains independent of *v* until a critical sweep rate *v*_o_ is reached (where the kinetic behavior is radically different), and then increases with log *v*.^85^ For example, with an increase in the difference between the cathodic and anodic peak voltage (Δ*E*_a,c_) as *v > v*_o_, a transition from reversible to irreversible kinetic processes occurs. And cyclic voltammograms are no longer mirror images of each other (as shown in Figure 5c,d).^83^, ^84^ However, the kinetic reversibility can be regained for a pseudocapacitor by lowering the sweep rate, since no phase change occurs during the charge/discharge processes. The presence of a kinetic limitation (characterized by the *v*_o_ value) determines the effective charge/discharge rates or power performance of ECs.

**Figure 6:**
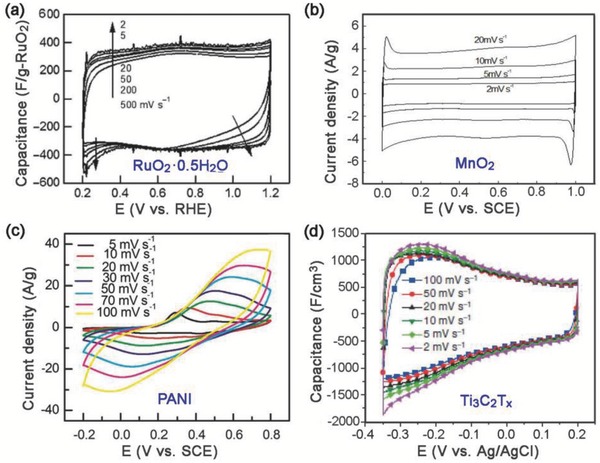
Typical CV profiles of: a) hydrous RuO_2_. Reproduced with permission.^92^ Copyright 2005, American Chemical Society. b) MnO_2_. Reproduced with permission.^93^ Copyright 2014, ESG. c) Polyaniline (PANI). Reproduced with permission.^95^ Copyright 2014, Elsevier Ltd. d) Mxene Ti_3_C_2_T*_x_* with different sweep rates. Reproduced with permission.^82^ Copyright 2014, Nature Publishing Group.

The cyclic voltammograms of EDLCs always exhibit a rectangular shape with no or little deviation upon increasing sweep rate, while those of pseudocapacitors can be rectangular, with or without cathodic/anodic peaks (or wide bumps), depending on the type of electrode material. Conductive polymers including polyaniline,^86^ polypyrrole, and PEDOT,^87^ and transition metal oxides involving MoO_3_,^88^ V_2_O_5_,^89^ T‐Nb_2_O_5_,^90^ tend to have broad redox peaks (**Figure** **6**c). Hydrous RuO_2_ 91, 92 (Figure 6a) and MnO_2_ 93 (Figure 6b) have been extensively investigated and exhibit nearly ideal rectangular CV curves without distinct redox peaks in aqueous electrolytes. Recently, cyclic voltammograms of Ti_3_C_2_T*_n_* Mxene were found to have a rectangular shape in sulfuric acid, resulting from the continuous change in the titanium oxidation state during charge/discharge processes (Figure 6d).^82^, ^94^ The CVs of these electrode materials demonstrate that the reversible faradaic reactions can exhibit similar electrochemical behavior compared to EDL capacitors. Hence, it is rather difficult to differentiate these two charge storage mechanisms from each other, especially for electrode materials that possess both EDL and pseudocapacitive mechanisms. To date, great efforts have been made to distinguish and estimate the contribution from these two surface‐controlled processes.

**Figure 7:**
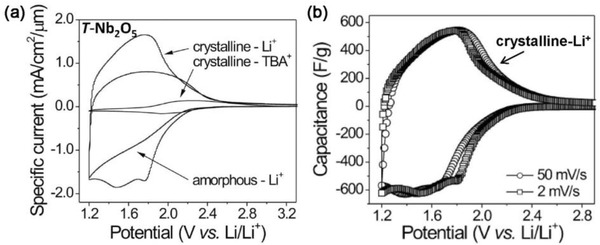
a) Cyclic voltammetry curves of amorphous and crystalline mesoporous T‐Nb_2_O_5_ films in lithium (Li^+^) and tetrabutylammonium (TBA^+^) electrolytes at a sweep rate of 10 mV s^−1^. b) Potential‐dependent capacitance calculated from CV curves at sweep rate of 2 and 50 mV s^−1^. Reproduced with permission.^96^ Copyright 2010, American Chemical Society.

Dunn and co‐workers^96^ demonstrated that CV can be used to estimate the contributions from the two charge storage mechanisms mentioned above through appropriate experimental design. Mesoporous Nb_2_O_5_ with crystallographically orientated layered nanocrystalline walls shows intercalation pseudocapacitance, because guest ions can be easily intercalated into the layers due to the weak van der Waal force between them. When such crystalline mesoporous Nb_2_O_5_ is used as an electrode, the charge storage mechanism can be altered by changing the electrolyte (e.g., the guest cations). When tetrabutylammonium (TBA^+^) perchlorate carbonate is added to the electrolyte, the charge storage changes to the EDL mechanism only, according to the CV curve shown in **Figure** **7**a. Since the bulky TBA^+^ cannot quickly intercalate into the layered Nb_2_O_5_. However, both EDL and intercalation pseudocapacitance contribute to the total charge storage in the LiClO_4_ electrolyte. And a much larger response current is identified than that of the EDL mechanism alone (Figure 7a). Thus, the relative contribution of EDL capacitance and pseudocapacitance can be estimated from CV results. Only a small fraction (≈10%) of the total charge results from EDL capacitance, while the majority is related to the surface confined faradaic charge transfer process. This is further confirmed by the broad redox peaks in the CV curves (Figure 7b), which is characteristic of a surface‐confined charge transfer process.^80^ However, it must be emphasized that the experimental method discussed here has many limitations, including the types and morphology of the electrodes. Hence, this method can only provide a rough estimation.

#### 2.2.2 Constant Current Charge/Discharge Curves

The profile of the potential versus capacitance for an EDL capacitor is a well‐defined linear shape, as described by Equations (2) and (3) and illustrated in Figure 3b. For pseudocapacitors, the electrode potential associated with the electrosorbed species is a continuous logarithmic function over the extent of sorption (Equation (8)), differs from the linear behavior of the EDL capacitor. In a constant current charge/discharge process, this translates into smooth charge/discharge profiles without pronounced plateaus (Figure 3d). In contrast, battery electrodes always deliver distinct charge/discharge potential plateaus in potential versus capacity profiles (Figure 3f), accompanying the phase transformations.

## 3 Quantifying the Capacitive Properties

Electrochemical analysis of different kinetic responses promotes better understanding of the charge/discharge mechanism, and provides basic guidance for the identification and design of high‐performance electrode materials for advanced energy storage devices. We summarize this analysis into three main approaches for distinguishing surface or bulk charge storage behavior and pseudocapacitive or battery‐type electrode materials in a quantitative way: (i) investigating difference of the redox (anodic (a) and cathodic (c)) peak potentials (Δ*E*_a,c_), (ii) establishing the relationship between the response current (*i*) and the sweep rate (*v*), and (iii) quantifying the relative contribution (%) of the capacitive and diffusion‐limited processes. These three methods are described in more detail in the following sections.

### 3.1 Redox Peak Difference (Δ*E*_a,c_)

**Figure 8:**
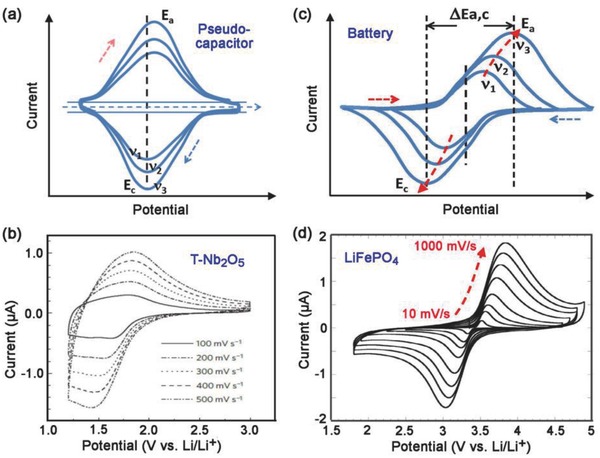
Typical CV curves of a) pseudocapacitive material and c) battery‐type material at different sweep rates, *v*_1_ < *v*_2_ < *v*_3_. Note that *v*_3_ < *v*_0_ for a pseudocapacitor and Δ*E*_a,c_ increases with increasing *v* for the battery. Typical experimental data for b) T‐Nb_2_O_5_. Reproduced with permission.^100^ Copyright 2013, Nature Publishing Group. d) LiFePO_4_. Reproduced with permission.^101^ Copyright 2011, The Electrochemical Society.

In CV measurements, the difference between the anodic and cathodic peak positions, Δ*E*_a,c_, and the voltage shift of the anodic and cathodic peaks with changing *v*, generally define the level of the reversibility of the electrochemical reactions.^97^, ^98^, ^99^ Pseudocapacitors have good reversibility and hence, Δ*E*_a,c_ is generally very small for these materials (Figures 3c and 8a,b) and remains constant over a wide range of *v* values until the critical sweep rate is reached (as described in Section 2.2.1).^24^ On the other hand, a large Δ*E*_a,c_ is generally observed for battery‐type materials that undergo crystallographic phase transformation during the electrochemical processes, even at a very low sweep rate (Figures 3e and 8c,d).^18^, ^23^ Typically, Δ*E*_a,c_ increases with increasing *v*, resulting in a poor rate performance of the battery (Figure 2a). Therefore, broad redox peaks in CV data can indicate pseudocapacitive behavior when Δ*E*_a,c_ is small or remains constant over a wide range of sweep rates (**Figure** **8**).^18^, ^23^

### 3.2 Relationship between the Response Current and Sweep Rate

**Figure 9:**
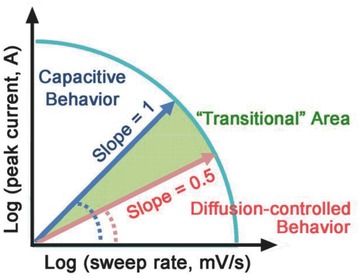
Power law dependence of the peak current on sweep rate (from Equation (12)) for capacitive materials (*b* = 1.0) and typical battery‐type materials (*b* = 0.5). The “transition” area between capacitive and battery‐type materials area is located in the range of *b* = 0.5–1.0.

**Figure 10:**
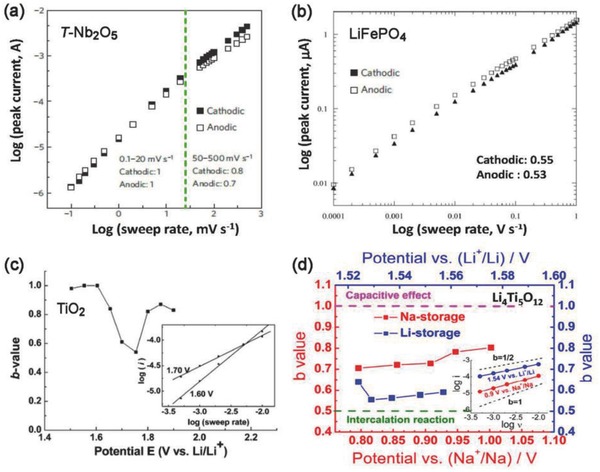
The dependence of parameter *b* on: a) electrode material types pseudocapacitive T‐Nb_2_O_5_. Reproduced with permission.^100^ Copyright 2013, Nature Publication Group. b) Battery‐type LiFePO_4_. Reproduced with permission.^101^ Copyright 2011, The Electrochemical Society. c) Potential (10 nm TiO_2_ film). Reproduced with permission.^103^ Copyright 2007, American Chemical Society. d) Charge storage mechanism (Li^+^ and Na^+^ reactions in Li_4_Ti_5_O_12_). Reproduced with permission.^109^ Copyright 2014, American Chemical Society. The inset in (c) shows the good linear dependence of the current on the sweep rate (based on Equation (12)) at 1.60 V (*b* = 1.0) and cathodic peak 1.70 V (*b* = 0.55). The inset in (d) shows the good linear dependence of the current on the sweep rate (based on Equation (12)) measured at the cathodic peak near a potential of 0.9 V for the Na^+^ storage case and 1.54 V for Li^+^ storage case.

In CV measurements, the total current measured under a potential sweep rate can be interpreted as the sum of the current related to the slow diffusion‐controlled process (*i*_diff_) and the current required to charge the double layer at the electrolyte interface or to initiate fast faradaic reactions on the exposed electrode surface (*i*_cap_).^102^ An empirical description of this is

(11) $$i\;\left(v\right)=i_{\mathrm{cap}}\;+\;i_{\mathrm{diff}}=av^{b}$$

(12) $$\log i\left(v\right)=\log a\;+b\log v$$

where both *a* and *b* are adjustable parameters.^101^, ^103^, ^104^, ^105^ Parameter *b* is determined from the slope of the linear plot of log *i* versus log *v* and is used to provide kinetic information about the electrochemical reactions (Equation (11)). There are generally two well‐defined conditions, *b* = 0.5 and *b* = 1 (**Figure** **9**). A *b* value of 1 signifies contributions from fast near‐surface activities, such as the fast surface redox reactions and charging/discharging the EDLCs. Thus, *i* (*v*) = *i*_cap_ = *av*, and the response peak current varies linearly with the sweep rate. While a *b* value of 0.5 represents the slow semi‐infinite diffusion‐controlled faradaic processes that occur in the bulk, such as battery‐type processes.^100^, ^101^, ^103^, ^104^ Therefore, by establishing the relationship between the peak current response and the sweep rate to estimate the value of *b*, the material type (pseudocapacitive or battery) can be identified and surface‐controlled processes (such as in EDLCs and pseudocapacitors) can be differentiated from diffusion‐controlled processes (i.e., in battery‐type electrodes). For example, *b* ≈ 1.0 was determined for the pseudocapacitive material, Nb_2_O_5_, (**Figure** **10**a) based on Equation (11),^100^ whereas a typical battery‐type material, LiFePO_4_, showed a *b* value ≈ 0.5 (Figure 10b),^101^ over a wide range of sweep rates.

The range of *b* values from 0.5 to 1.0 indicates a “transition” area between pseudocapacitive materials and battery‐type materials, though a clear boundary is not easy to define. Generally, the smaller the *b* value is, the larger the contribution from diffusion‐controlled intercalation processes, while the capacitive contribution increases with increasing *b* value. Typical examples include the insertion of Li^+^ into TiO_2_ films (Figure 10c),^103^, ^106^, ^107^ Na^+^ intercalation into TiO_2_/graphene nanocomposites,^108^ and Li^+^/Na^+^ reaction with Li_4_Ti_5_O_12_ (Figure 10d).^109^ It was found that the *b* value was strongly dependent on the potential, sweep rate, and charge storage mechanism, in addition to the material type. For example, a *b* value of 0.55 at the cathodic peak potential of 1.70 V was calculated for anatase TiO_2_ films, indicating that the Li^+^ intercalation reaction dominated the current.^103^ Away from the peak potential, *b* increased to 0.8–1.0, suggesting that the current primarily stemmed from capacitive contribution. The presence of a critical sweep rate, as discussed in Section 2.2.1, results in a change in *b* value with the sweep rate. As illustrated in Figure 10a, the *b* value of Nb_2_O_5_ remains around 1 up to 50 mV s^−1^, and then decreases when the sweep rate increases further, indicating the kinetics transition from surface‐controlled behavior to semi‐infinite linear diffusion.^100^ Similar phenomenon has also observed in other electrode materials.^108^ In addition, different charge storage mechanisms also result in different *b* values (Figure 10d).^109^ For Li^+^ storage in Li_4_Ti_5_O_12_,^109^ *b* values in the range of 0.55–0.65, close to 0.5, were observed, indicating that the charge storage primarily resulted from diffusion‐controlled intercalation processes with well‐defined redox peaks and a distinct charge/discharge plateau. While for the Na^+^ storage case, the *b* values were in the range of 0.7–0.8,^109^ higher than that of Li^+^ storage. This suggests a higher capacitive contribution as a result of the larger ionic size of Na^+^ than that of Li^+^. This difference is also manifested in broader CV peaks and a poorly defined discharge plateau for Na^+^ storage compared with Li^+^ storage.

The *b* value can be used to provide guidance for the practical design of high‐performance electrode candidate materials. For example, the *b* value could (i) act as an indicator for differentiating pseudocapacitive from battery‐type materials, and (ii) provide more kinetic information about electrochemical reactions in terms of charge storage types at different potential/sweep rates and charge storage mechanisms for different ion intercalation batteries.

### 3.3 Differentiating Capacitive Effect from Diffusion‐Controlled Process

Based on the discussion of parameter *b* in Section 3.2, quantitatively distinguishing between capacitive processes and diffusion‐controlled intercalation processes is therefore highly desirable for a better understanding of the underlying charge storage mechanism to aid materials selection and device design.

When the process is controlled by surface‐dominant reactions, the response current varies linearly with *v* (i.e., d*i*/d*v* = constant = capacitance). If the process is controlled by semi‐infinite diffusion, the response current varies linearly with *v* ^1/2^ (i.e., d*i*/d*v* ^1/2^ = constant).^102^, ^110^ Thus, a general expression taking into account all possible cases is^102^, ^110^

(13) $$i\;\left(V\right)=i_{\mathrm{cap}}\;+\;i_{\mathrm{diff}}=k_{1}v+k_{2}v^{1/2}$$

or

(14) $$i\left(V\right)/v^{\frac{1}{2}}=\;\;\;k_{1}\;v^{\frac{1}{2}}+k_{2}$$

**Figure 11:**
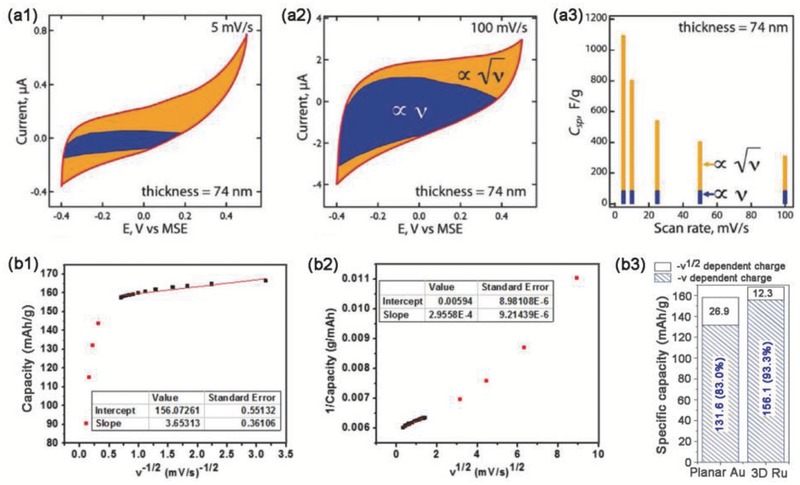
Two different methods for deconvoluting surface (∝*v*) and bulk charge (*v* ^1/2^): a1–a3) *I* ∝ *v* or *v* ^1/2^ and b1–b3) *q ∝ v* ^1/2^ or *v* ^−1/2^. CV curves at (a1) 5 and (a2) 100 mV s^−1^ for MnO_2_ (74 nm shell)‐Au (core) hierarchical structure. (a3) Dependence of surface/bulk charge ratio on sweep rate. Reproduced with permission.^111^ Copyright 2012, American Chemical Society. (b1) Gravimetric capacity versus *v* ^−1/2^ and (b2) inverse gravimetric capacity versus *v* ^1/2^ for V_2_O_5_/Ru nanotube arrays. The intercept value in (b1) represents the surface charge (∝*v*). The inverse of the intercept in (b2) is the total charge. (b3) Surface/bulk charge ratio for V_2_O_5_/planar Ru and V_2_O_5_/Ru nanotube array hybrids. Reproduced with permission.^112^ Copyright 2014, Nature Publication Group.

Using Equation (14), the constants *k*_1_ and *k*_2_ can be evaluated from the slope and intercept, respectively, of a linear plot of *i*(*V*)/*v* ^1/2^ versus *v* ^1/2^. Consequently, it is possible to quantitatively differentiate the current contribution from the capacitive effect (*k*_1_ *v*) from diffusion‐controlled intercalation processes (*k*_2_ *v* ^1/2^). As shown in **Figure** **11**a, the surface‐dominant capacity was estimated to be around 88 F g^−1^ for a MnO_2_/Au (shell–core) hierarchical nanostructure at a sweep rate of 5 mV s^−1^, and this value was nearly constant over a wide range of sweep rates (Figure 11a2,a3). The diffusion‐controlled insertion capacity was found to be sweep rate dependent, with the surface/bulk charge ratio increasing gradually.^111^

Another method used to differentiate capacitive and diffusion‐controlled processes is by establishing a relationship between the total stored charge and the sweep rate, as developed by Ardizzone et al.^113^ The total charge q* contains a capacitive contribution *q*_s_* (proportional to *v*) and a diffusion‐controlled contribution *q*_d_*(proportional to *v* ^1/2^)

(15) $$q^{*}\left(v\right)=q_{s}{}^{*}\;\;+q_{d}{}^{*}$$

The *q*_s_* value corresponding to surface‐dominant processes is obtained at an infinite potential sweep rate (*v* → ∞), where

(16) $$q^{*}\to \;q_{s}{}^{*}$$

The maximum charge density (*q*_m_*) is obtained when *v* → 0 and

(17) $$q^{*}\to q_{m}{}^{*}$$

In particular, q* is expected to be limited by *v* ^−1/2^ if semi‐infinite linear diffusion is involved, whereas capacitive contributions are independent of the sweep rate; thus, Equation (15) can be expressed as follows

(18) $$q^{*}\left(v\right)=q_{v\to \;\infty }{}^{*}\;\;\;\;\;\;\;+\;\;q_{d}{}^{*}\;\;=q_{v\to \;\infty }{}^{*}\;\;\;\;\;\;\;+\mathrm{const}\;{\left(v^{-1/2}\right)}^{*}$$

where the minimum *q*_s_* value is determined from the extrapolation of a linear plot of q* versus *v* ^−1/2^ to *v* ^−1/2^ = 0. Generally, q* decreases as *v* increases because of diffusion limitations.^113^ Since q* increases linearly with *v* ^−1/2^, it is reasonable to expect that 1/q*(*v*) decreases linearly with *v* ^1/2^

(19) $$1/q^{*}\left(v\right)=1/q_{m}{}^{*}\;\;\;\;+\;\;\;\mathrm{const}\;{\left(v^{1/2}\right)}^{*}$$

where *q*_m_* is the maximum total charge. Extrapolation of a linear plot of 1/q*(*v*) versus *v* ^1/2^ to *v* ^1/2^ = 0 gives the basic amount of the maximum total charge *q*_m_*. Consequently, the difference between the total charge (*q*_m_*) and the surface charge (*q*_*v*→∞_*) gives the charge associated with the diffusion‐controlled processes

(20) $$q_{d}{}^{*}\;\;=q_{m}{}^{*}\;\;-q_{v\;\to \;\infty }{}^{*}$$

Therefore, the contributions from the capacitive and the semi‐diffusion controlled processes can be estimated. A typical example is the electrochemical behavior of V_2_O_5_ coated on Au tube arrays.^112^ The surface‐dominant and total charges were estimated to be 156.1 mA h g^−1^ from a *C* versus *v^−^* ^1/2^ plot (Figure 11b1) and 168.4 mA h g^−1^ from a 1/*C* versus *v* ^1/2^ plot (Figure 11b2), respectively. Moreover, the bulk charge can be efficiently increased via a 3D current collector design, as illustrated in Figure 11b3. This opens a new opportunity for achieving high power/energy density electrode materials for advanced energy storage devices.

## 4 Optimizing Pseudocapacitive Electrode Design

The methods discussed in Section 3 for quantitatively differentiating the two charge storage mechanisms can be used to identify high‐performance intrinsic electrodes, explore extrinsic electrode behavior, and design novel hybrid electrodes.

Materials including mesoporous α‐MoO_3_,^114^ TiO_2_,^103^, ^108^, ^115^ Nb_2_O_5_,^100^ hierarchical MnO_2_,^116^ and Li_4_Ti_5_O_12_ 109 have been well described using Equations (11) and (12). It is not surprising that the contribution from the two different processes (capacitive and diffusion‐controlled) is strongly dependent on the structure, crystallinity, and morphology of the electrode materials,^96^, ^103^, ^114^, ^116^ type of electrolyte,^96^, ^116^ sweep rate,^116^ and charge storage mechanism.^109^ Generally, the capacitive contribution dominates the total current response or charge storage for EDL and pseudocapacitive electrodes. Therefore, the contribution ratio of the capacitive versus diffusion‐limited process may be another effective indicator for differentiating pseudocapacitive materials from battery‐type candidates.

### 4.1 Intrinsic Pseudocapacitive Materials

Any electrode material exhibiting linear or approximately linear charge/discharge curves without a pronounced voltage plateau and delivering broad and nearly overlapped redox couple peaks in CV curves, can be regarded as a pseudocapacitive material. Pseudocapacitive behavior can be intrinsic or extrinsic, depending on the nature of the electrode material and materials engineering undertaken. Intrinsic pseudocapacitive materials possess typical characteristics of capacitive charge storage, regardless of their crystalline properties, morphology, or particle size. Typical intrinsic pseudocapacitive materials include MnO_2_,^70^, ^117^, ^118^ RuO_2_,^119^, ^120^ and various conducting polymers such as polypyrrole,^121^, ^122^ polyaniline,^64^, ^123^ and PEDOT.^124^, ^125^ In addition, other pseudocapacitive materials, such as TiO_2_ (B), α‐MoO_3_, T‐Nb_2_O_5_,^100^ and Li_4_Ti_5_O_12_,^109^ have been identified based on the quantitative differentiation of the capacitive effect from the diffusion‐controlled process.

#### 4.1.1 TiO_2_ (B) and Hydrogen Titanates

**Figure 12:**
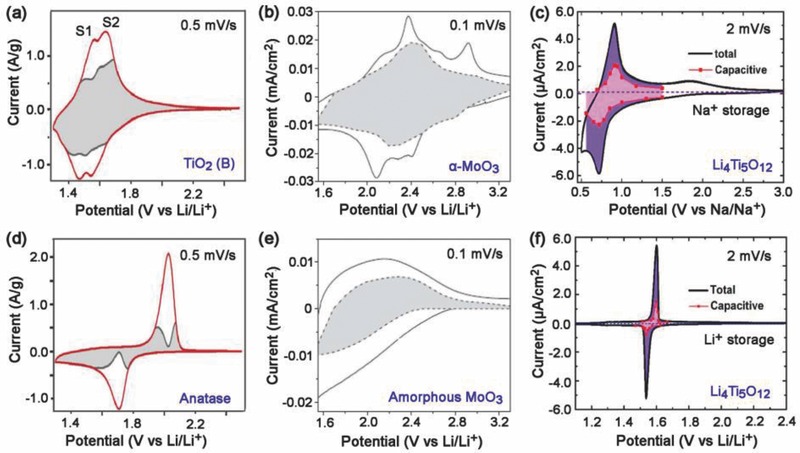
The dependence of intrinsic pseudocapacitive behavior on: crystal structure a) TiO_2_ (B) and d) anatase TiO_2_. Reproduced with permission.^107^ Copyright 2014, Elsevier Ltd. b) a‐MoO_3_ and e) amorphous MoO_3_. Reproduced with permission.^114^ Copyright 2010, Nature Publication Group. and charge storage mechanism c) Na^+^ storage and f) Li^+^ storage. Reproduced with permission.^109^ Copyright 2014. American Chemical Society.

TiO_2_ (B) is a metastable modification of titanium dioxide with the monoclinic structure (space group C2/m; lattice parameters *a* = 1.21787 nm, *b* = 0.37412 nm, *c* = 0.65249 nm, and β = 107.054°) that is characterized by two edge‐sharing TiO_6_ octahedra linked to a neighboring pair of octahedra by their corners.^106^ This material has a low density (3.64–3.76 g cm^−3^) and an open structure, which is particularly suitable for pseudocapacitive energy storage.^106^, ^126^ The pseudocapacitive behavior of TiO_2_ (B) is characterized by a dominant pseudocapacitive faradaic process via Li^+^ intercalation. Two pairs of redox peaks located at 1.5 and 1.6 V have been detected in the CV curve, accompanying the insertion of Li^+^ into the TiO_2_ (B) lattice.^107^ Note that these potentials are lower than that for Li^+^ intercalation into anatase TiO_2_ (≈1.70 V) and the redox peaks are broader, indicating that a different charge storage mechanism is involved. A linear relationship between the peak current and sweep rate was observed for TiO_2_ (B), verifying that the accommodation of Li into the TiO_2_ (B) is dominated by the capacitive effect. The capacitive contribution was calculated to be 68% of the total charge for TiO_2_ (B) (**Figure** **12**a), two times that of anatase TiO_2_ (34%, Figure 12d), in spite of the former having a surface area three times smaller than the latter.^107^ The total stored charge was 625 C g^−1^ for pure TiO_2_ (B) at a sweep rate of 0.5 mV s^−1^, which was 27% higher than that for anatase (Figure 12d). The pseudocapacitive behavior of TiO_2_ (B) is ascribed to the open structure allowing fast Li^+^ transport in the bulk TiO_2_ (B) lattice along the *b*‐axis.

Hydrogen titanates, primarily H_2_Ti_3_O_7_, have also shown pseudocapacitive behavior, characterized by broad redox peaks and the liner dependence of the peak current on the sweep rate.^126^, ^127^, ^128^, ^129^, ^130^ Its layered structure consisting of zigzag ribbons of edge‐sharing TiO_6_ octahedra provides an open‐layered framework to facilitate Li^+^ insertion. Note that hydrogen titanates undergo consecutive phase changes with increasing temperature: TiO_2_ (B) at 400 °C, anatase at 700 °C, and rutile at 1000 °C.^131^ The capacitive effect of hydrogen titanates is therefore dependent on the annealing temperature and resulting morphology.^132^, ^133^, ^134^ A transition from pseudocapacitive behavior of the protonated titanate to coexisting pseudocapacitive and diffusion‐limited behavior of the TiO_2_ (B) and anatase TiO_2_ mixture, to the diffusion‐limited behavior of anatase TiO_2_ has been well studied.^107^, ^133^

#### 4.1.2 Orthorhombic MoO_3_ and Nb_2_O_5_

Orthorhombic MoO_3_ (α‐phase) is another promising pseudocapacitor electrode material with an advantageous unique layered structure along the [010] direction. The double layers of the MoO_6_ octahedra are bonded by covalent forces in the^100^ and [001] directions and by Van der Waals forces in the [010] direction.^88^, ^135^ The weakly bonded interlayers are particularly desirable for ion intercalation and transport, and result in pseudocapacitance behavior.^136^ The corresponding charge storage in MoO_3_ occurs due to (i) redox pseudocapacitance arising from charge‐transfer processes across the interface and (ii) intercalation pseudocapacitance resulting from ion intercalation into van der Waals gaps.^136^ The pseudocapacitive behavior is strongly dependent on the crystalline structure. Dunn and co‐workers^114^ found that the capacitive contribution could be improved significantly for mesoporous α‐MoO_3_ (Figure 12b, 70% of the total charge storage) compared to amorphous materials (Figure 12e, 35%). The capacitive charge storage was 450 C g^−1^ for the crystalline mesoporous film, three times that of amorphous films (150 C g^−1^). Moreover, the crystalline films delivered higher total charge storage and a faster charging/discharging rate than the amorphous films. This is attributed to the extra Li^+^ intercalation pseudocapacitance in mesoporous α‐MoO_3_ films due to the iso‐oriented crystal structure with preferred intercalation planes.^114^ Dunn and co‐workers^100^ also discovered that Li ions intercalation into T‐Nb_2_O_5_ possess similar trend with α‐MoO_3_. The kinetics of charge storage is also influenced significantly by crystallization.^137^ At charging time of only 12 s, the capacity is ≈450 C g^−1^, and achieves a consistent value of 560 C g^−1^ as time increases to 2 min; thus, over 80% of the capacity is accessed within 12 s, indicating the ultrafast kinetic response. The reason is that the mostly empty octahedral sites between (001) planes provide natural tunnels for fast diffusion kinetics in the *a*‐*b* plane. These results demonstrate how improved pseudocapacitance can be achieved via the design of unique iso‐oriented crystalline mesoporous structures. Hence, gaining insight into the charge storage mechanisms in different crystalline structure is another effective method for selecting high‐performance electrode materials.

#### 4.1.3 Li_4_Ti_5_O_12_

Spinel‐type Li_4_Ti_5_O_12_ (LTO), is a promising “zero‐strain” anode material for lithium‐ion battery that experiences a two‐phase reaction (Li_4_Ti_5_O_12_/Li_7_Ti_5_O_12_) and shows a well‐defined voltage plateau (1.55 V vs Li/Li^+^).^138^, ^139^ It was found that sodium ions can also be reversibly inserted/extracted from the Li_4_Ti_5_O_12_ lattice, in spite of Na^+^ having a larger ionic radius (0.102 nm) than that of Li^+^ (0.076 nm).^140^, ^141^ In contrast to the two‐phase reaction in the Li^+^ intercalation/de‐intercalation process, a three‐phase reaction (2Li_4_Ti_5_O_12_ + 6Na^+^ + 6e^−^ ↔ Li_7_Ti_5_O_12_ + Na_6_LiTi_5_O_12_) is observed during Na^+^ intercalation.^141^ This different ion charge storage mechanism is also characterized by the pseudocapacitive behavior for Na^+^ storage in Li_4_Ti_5_O_12_ in terms of broad redox peaks with small peak separation in CV curves (Figure 12c) and indistinct voltage plateaus in charge/discharge curves. A capacitive contribution of 51% of the total charge for Na^+^ storage was observed, which is two times higher than that for Li^+^ storage (24%, Figure 12f).^109^ The pseudocapacitive behavior of Na^+^ storage in Li_4_Ti_5_O_12_ depends on the particle size^142^ and film thickness.^109^

Li_4_Ti_5_O_12_ is a typical battery‐type material for Li^+^ storage, but pseudocapacitive for Na^+^ storage. Therefore, Li_4_Ti_5_O_12_ exhibits intrinsic intercalation pseudocapacitive behavior in nonaqueous electrolytes. Traditional battery‐type materials for Li^+^ storage can be pseudocapacitive when different guest ion intercalation processes take place in different electrochemical systems. This is of particular interest for designing high‐power energy storage devices based on traditional high‐energy density materials via introducing different guest ion intercalation reactions.

#### 4.1.4 MXenes

MXenes, a new class of 2D stacked materials, are emerging as promising candidates for electrodes in electrochemical energy storage applications, such as supercapacitors and batteries, due to their good conductivity and a broad range of chemistries.^81^, ^143^, ^144^ MXenes are produced by the selective etching of the A‐group (generally group IIIA and IVA elements) layers from the ternary transition metal carbide (MAX phases, e.g., Ti_3_AlC_2_, Ti_2_AlC, and Ta_4_AlC_3_) via wet chemistry routes.^82^, ^145^, ^146^ MXenes combine the metallic conductivity of transition metal carbides with the hydrophilic nature of their hydroxyl or oxygen‐terminated surface. These features are of great interest for supercapacitor applications. An impressive high volumetric capacitance (900 F cm^−3^, comparable with hydrated RuO_2_) was demonstrated in aqueous electrolytes.^82^, ^147^ In situ X‐ray absorption spectroscopy (XAS) revealed continuous changes in the Ti oxidization state during charge/discharge cycling.^94^ Variations in the distance between the Ti_3_C_2_T*_x_* layers (*c*‐axis) due to cation insertion/de‐insertion was estimated to be <5% using in situ X‐ray diffraction, and no phase changes were detected.^81^ It seemed that the capacitive charge storage resulting from cationic intercalation/de‐intercalation occurred so rapidly that it resembled 2D ion adsorption at solid–liquid interfaces.^144^, ^148^ Surface capacitive effects, either electrostatic or pseudocapacitive, dominate charge storage in these materials.^82^, ^144^ These findings validate the intrinsic pseudocapacitive nature of MXenes, expand the family of pseudocapacitive materials, and provide new insights for designing high‐performance electrode materials.

### 4.2 Extrinsic Pseudocapacitive Materials

Nanostructured materials for EES offer the unique opportunity of tailoring the energy and power density and enabling operation in the intermediate stage between battery and EC behavior. The optimization of traditional battery‐type electrode materials on the nanoscale has yet to be realized, but it is an exciting direction for increasing their power density because of the short ion and electron transport paths.^149^ However, the electrochemical responses of traditional battery‐type electrodes are strongly dependent on the particle size and morphology of the electrode material.

**Figure 13:**
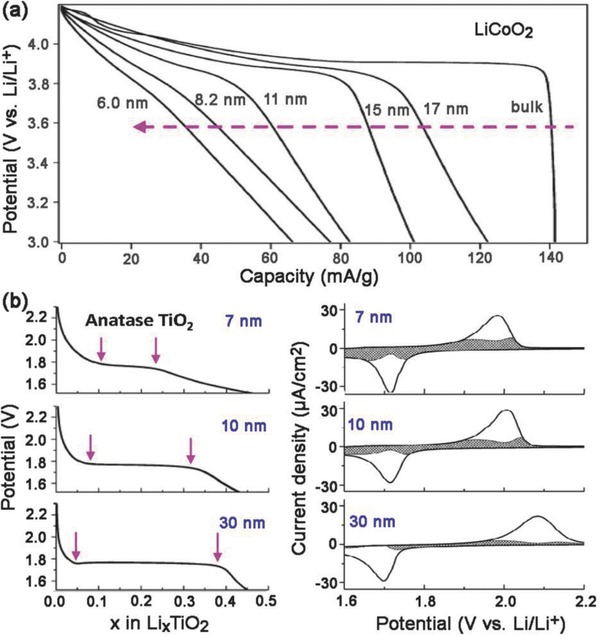
Dependence of the extrinsic pseudocapacitive behavior on crystallite size for a) LiCoO_2_. Reproduced with permission.^152^ Copyright 2007, American Chemical Society. b) Anatase TiO_2_. Reproduced with permission.^103^ Copyright 2007, American Chemical Society.

One typical example is LiCoO_2_, which is a commercial cathode material for lithium‐ion batteries. The bulk LiCoO_2_ exhibits a well‐defined discharge plateau at about 3.9 V and well‐separated redox peaks in CV curves.^150^, ^151^ However, the discharge plateau region (the capacity from the inner layers) decreased gradually and capacitor behavior (the capacity from the intercalation of Li^+^ ions into the surface layers) became more dominant with decreasing crystallite size (**Figure** **13**a).^152^ In particular, a nearly linear discharge curve was observed when the particle size reduced to 6 nm, verifying the transition from battery‐type to pseudocapacitive behavior.

Similar trends have also been observed for anatase TiO_2_, which is another typical battery‐type material with a Li^+^ insertion potential plateau at about 1.75 V (Figure 13b).^103^, ^153^ The constant‐voltage region (indicated by the arrows on the figure) that represents a two‐phase reaction during the Li^+^ insertion was much shorter for 7 nm particles (*x* ≈ 0.15) than that of 30 nm particles (*x* ≈ 0.35), where *x* is the Li content in Li*_x_*TiO_2_.^103^ The transition from battery‐type to pseudocapacitive behavior is exhibited by a gradual increase in the capacitive contribution and a decrease in the peak potential difference (Δ*E*_a,c_) with decreasing particle size. The capacitive contributions were 15, 35, and 55% of the total Li^+^ storage for 30, 10, and 7 nm particle sizes, respectively.^103^ It is clear that capacitive behavior becomes dominant with decreasing crystallite size.

The typical well‐defined flat discharge curve of a bulk material changes to a sloped curve for nanostructured materials. This is due to the emergence of extrinsic faradaic reactions on the surface or near‐surface region that replace diffusion‐controlled lithium ion interactions when a battery‐type material is engineered to be nanosized (with a large surface area and short ion diffusion distance). Therefore, a superior rate performance has been reported for nanocrystals compared to bulk battery‐type materials. Nanosized battery‐type materials, such as V_2_O_5_ 104 and CeO_2_,^154^ also show extrinsic pseudocapacitive behavior. Materials that exhibit pseudocapacitive behavior on the nanoscale but battery‐type behavior in the bulk state are therefore denoted as extrinsic pseudocapacitive materials.^18^, ^23^

### 4.3 Hybrid Materials

It should be noted that most of the faradaic electrodes that can provide surface redox capacitance or ion intercalation show poor electronic conductivity. The rational design of electrode materials with fast charge‐transfer kinetics in the surface or bulk is therefore highly desirable. There are several ways to achieve this, such as decreasing the size or producing a hybrid with highly conductive materials. The first approach involves engineering nanoscale electroactive materials with different morphologies (nanoparticles, rods, sheets, branches, etc.) to shorten the ion diffusion length (as discussed in Section 4.2). The second approach focuses on enhancing the electrochemical performance by maximizing the coupling effects of their individual components. For the sake of simplicity, the components of a hybrid electrode are classified as a conductive part (i.e., various carbon allotropes, metals) and an electrochemically active part (i.e., pseudocapacitive and battery‐type parts).

#### 4.3.1 Binary Hybrids

**Figure 14:**
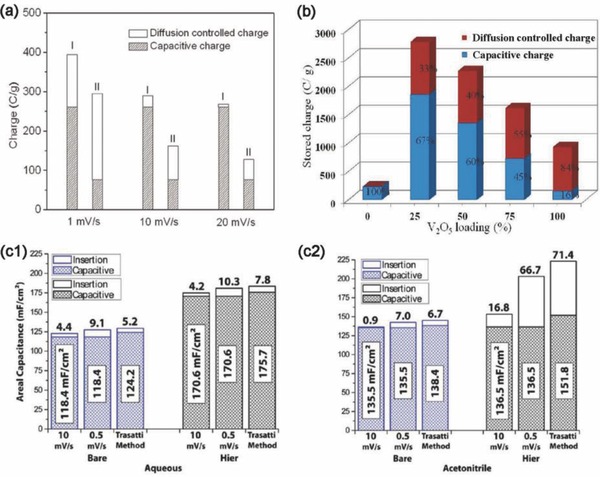
Characterization results of binary hybrids. The dependence of capacitive charge and diffusion‐controlled charge on the hybrid material a) I is V_2_O_5_/CNT and II is V_2_O_5_. Reproduced with permission.^155^ Copyright 2012, American Chemical Society. b) V_2_O_5_/CNT hybrids with different V_2_O_5_ mass loadings. Reproduced with permission.^104^ Copyright 2011, American Chemical Society. And electrolyte type c1) aqueous 1 m LiClO_4_ and c2) organic acetonitrile 1 m LiClO_4_. Reproduced with permission.^116^ Copyright 2013, American Chemical Society.

The design of hybrid materials, which combine electroactive and conducting components in a single electrode, can offset the slow charge‐transfer kinetics originating from the low electrical conductivity of the electroactive materials. For example, a V_2_O_5_ nanowire/CNT hybrid (sample I, **Figure** **14**a) exhibits enhanced pseudocapacitive behavior in terms of the specific capacitance and the rate capability compared to pure V_2_O_5_ nanowires (sample II, Figure 14a) in NaClO_4_/propylene carbonate (PC).^155^ This better performance was due to the high electrical conductivity (≈3.0 S cm^−1^) and the hierarchical porosity of the intertwined networks.^155^ In particular, when pure V_2_O_5_ nanowires were used as the electrode, the total charge came mainly from the diffusion‐controlled process. In the case of the V_2_O_5_/CNT nanocomposite, the capacitive contribution dominated the total charge storage at all sweep rates, indicating that most of the Na^+^ intercalation sites were available in V_2_O_5_ within the nanocomposite. Moreover, a decrease in total stored charge and a transition from capacitive to battery‐type behavior was observed with increasing V_2_O_5_ fraction in the V_2_O_5_/CNT composites (Figure 14b),^104^ resulting from a decrease in overall surface area and degradation in electrical conductivity. Similar results were observed for MnO_2_/Au (shell/core) nanowires with different MnO_2_ thicknesses.^111^ Consequently, the design of high‐performance hybrid electrodes with the optimum combination of capacitive and diffusion‐controlled charge storage is possible via the optimization of mass loading or thickness of the electroactive materials in the hybrid electrode.

Another hybridization approach for optimizing the electrode design, combining two electroactive constituents to form a hierarchical structure in a single electrode, has also been demonstrated.^116^ This is equivalent to the parallel combination of faradaic materials, thus increasing the total stored charge. For example, a hierarchical structure consisting of individual MnO_2_ nanowires surrounded by a conformal layer of MnO_2_ nanofibrils showed enhanced area‐specific capacitance compared to bare MnO_2_ nanowires in both aqueous and organic electrolytes, as shown in Figure 14c1,c2.^116^ The increase in capacitive charge storage dominated the increase in total capacitance in the aqueous electrolyte. Meanwhile, the increase in capacitance using the organic electrolyte mainly resulted from the increase in diffusion‐controlled charge storage. The different charge storage mechanism was attributed to the proton adsorption behavior in water that mitigates the lithium intercalation mechanism. A synergetic effect was also identified in other hierarchical structures with different electroactive materials, including H‐TiO_2_/MnO_2_,^156^, ^157^ MnO_2_/polyaniline,^158^ MoO_3_/polypyrrole,^159^ MoO_3_/TiO_2_,^160^ V_2_O_5_/polypyrrole,^161^ and V_2_O_5_/PANI.^162^ Such hierarchical designs could combine the advantages of the two components in terms of providing a large accessible surface area for charge transfer and a shorter diffusion path for insertion/extraction of active species, thus improving electrochemical performance compared to the individual components.

#### 4.3.2 Going beyond Binary Hybrids

Ternary hybrid structures have been explored in order to take advantage of the different merits of the components (conductive additives, pseudocapacitive metal oxides, and/or conducting polymers). One typical ternary electrode composed of MnO_2_, CNTs, and PEDOT‐PSS demonstrated significant improvement in the electrochemical performance.^163^ Each component in the MnO_2_/CNTs/PEDOT‐PSS hybrid structure contributed to the improved electrochemical properties. The MnO_2_ nanospheres provided high specific capacitance, the CNTs offered high surface area for the deposition of MnO_2_ and provided good electrical conductivity and mechanical stability, and the PEDOT‐PSS acted as an effective dispersant for the MnO_2_/CNTs composite, and a good conductive binder for ensuring good electric contact between the MnO_2_ nanoparticles and CNTs. In addition, PEDOT‐PSS is also an electroactive material that provided extra pseudocapacitance. Such synergistic effects were also identified in other alternative ternary structures, for example, MnO_2_/graphene/CNTs, MnO_2_/graphene/PEDOT‐PSS,^164^ and MnO_2_/graphene/PANI.^165^ The specific capacitance of these electrode were significantly increased by around 20 and 45%, by 3D conductive wrapping of MnO_2_/graphene nanostructures with CNTs and PEDOT‐PSS, respectively.^164^ This ternary design takes advantage of the unique properties of each component, resulting in hybrid composites with high specific capacitance, good rate capability, and long cycle life. Such a strategy highlights a promising direction for optimizing pseudocapacitive materials that achieve high energy density and can be integrated into high‐performance energy storage devices.

## 5 Conclusions and Future Perspectives

The full potential of nanostructured capacitive materials, especially extrinsic pseudocapacitive materials, and hybrid electrodes has not yet been realized. The performance, in terms of the capacitance, rate capability, and cycle stability, needs to be further improved and a proper balance needs to be considered. However, some fundamental criteria for identifying potential high‐performance pseudocapacitive electrode materials have been proposed, along with strategies for hybrid electrode design. Intrinsic and extrinsic pseudocapacitive materials have been identified from both thermodynamic and kinetic point of view. Advanced approaches, aiming at introducing more electrochemically active sites and shortening the transport path for electrons and diffusion length for ions, have been discussed. This is achieved through the selection of an appropriate pseudocapacitive material and the careful design of the hybrid electrode architecture. Furthermore, the ability to quantitatively differentiate between the capacitive and diffusion‐controlled processes assists in tailoring the hybrid electrode for different applications.

There are several important points to consider regarding the topic of pseudocapacitive materials and hybrid electrodes:

(1) The same electrode material, depending on its structure, morphology, particle size, and guest ion, may display pseudocapacitive or battery‐like behavior. The identification of the types of such electrode materials through the quantitative differentiation between capacitive and diffusion‐controlled processes is critically important.
(2) The development of ternary hybrid structures is a promising direction for optimal electrode design as the positive properties of all components can be combined. A good understanding of surface chemical interactions between the components is essential to boost synergistic effects to enhance charge transfer and storage. This knowledge is extremely lacking for ternary hybrids, although the charge storage mechanism in binary structures has been well explained.
(3) Going beyond hybrid electrodes, hybrid energy storage devices consisting of a Faradaic battery‐type electrode and a Faradaic pseudocapacitive or a non‐Faradaic double layer electrode, or consisting of hybrid battery‐capacitor electrodes, could be promising alternatives to break the energy density limitation of traditional electrochemical capacitors and the kinetic limitation of batteries. The key challenge lies in a thorough understanding of the basic electrochemistry of the double hybridization approach. In addition, the selection of appropriate electrode materials and the design of unique hybrid electrodes are key factors in realizing the full potential of hybrid electrode materials and hybrid energy storage devices.

## Conflict of Interest

The authors declare no conflict of interest.
